# Generation of highly integrated multiple vivid colours using a three-dimensional broadband perfect absorber

**DOI:** 10.1038/s41598-019-49906-3

**Published:** 2019-10-16

**Authors:** Soo-Jung Kim, Pil-Hoon Jung, Wonjoong Kim, Heon Lee, Sung-Hoon Hong

**Affiliations:** 1 Department of Materials Science and Engineering, Korea University, Anam-dong 5-1, Sungbuk-Ku, Seoul 136–701 Republic of Korea; 2 ICT Materials and Components Research Laboratory, Electronic and Telecommunications Research Institute Gajeong-dong, Yuseong-gu, Daejeon 305–700 Republic of Korea

**Keywords:** Engineering, Materials science, Nanoscience and technology, Optics and photonics

## Abstract

The colour printing technology based on interactions between geometric structures and light has various advantages over the pigment-based colour technology in terms of nontoxicity and ultrasmall pixel size. The asymmetric Fabry–Perot (F–P) cavity absorber is the simplest light-interacting structure, which can easily represent and control the colour by the thickness of the dielectric layer. However, for practical applications, an advanced manufacturing technique for the simultaneous generation of multiple reflective colours is required. In this study, we demonstrate F–P cavity absorbers with micropixels by overcoming the difficulties of multi-level pattern fabrication using a nanoimprinting approach. Our asymmetric F–P cavity absorber exhibited a high absorption (approximately 99%) in a wide visible light range upon the incorporation of lossy metallic materials, yielding vivid colours. A high-resolution image of eight different reflective colours was obtained by a one-step process. This demonstrates the potential of this technology for device applications such as high-resolution colour displays and colour patterns used for security functions.

## Introduction

Structural colour obtained using Fabry–Perot (F–P) cavity structures^1–6^ has attracted considerable attention for the development of ink-free colour printing techniques for applications in anticounterfeiting devices^7–11^, functionalised colour decoration^12–16^, and reflective displays^17–20^. The colour spectrum exhibits enhanced colour saturation and brightness by the high reflectance peak and deep absorption valley through manufacturing by a simple deposition or coating. Furthermore, the colours can easily be changed by adjusting the thickness of the cavity layer. Recently, asymmetric F–P cavity structures based on highly lossy materials and porous materials^21–24^ have exhibited broadband absorption properties providing reflective vivid colours. However, it is still challenging to integrate multiple colours with a high resolution by varying the dielectric thickness on the same plane for practical applications. Furthermore, the lithography-based fabrication is very challenging and time-consuming, which significantly hinders the implementation of full-colour images of a large area^25,26^. To achieve monolithic integrated colours, a simple patterning process, such as soft-nanoimprint lithography^27–30^ and transfer printing^31^, is required to simultaneously fabricate various cavity layers.

In this study, we fabricated highly integrated pixelated F–P resonance absorbers with eight different cavity layers by a one-step nanoimprint process. Our asymmetric F–P cavity structure included a top film of porous metal, intermediate layers of dielectrics, and conventional metallic bottom mirror. A silver nanocrystal (Ag NC) film was employed as the metallic top layer, owing to its high optical loss in the visible light range. It could provide a relatively broad absorption band and thus better colour saturation and purity^32^, desirable for colour printing applications. Through the combination of the lossy porous metallic film and systematically designed multi-level-thickness dielectric layer, a microscopic colour artwork could easily be produced with a high resolution. The reflective colour pixels based on a multi-stepped layer are promising for full-colour printing applications.

## Results

### Numerical calculation and measurement of the colouration of the Ag-NC-based F–P absorber

**Figure 1:**
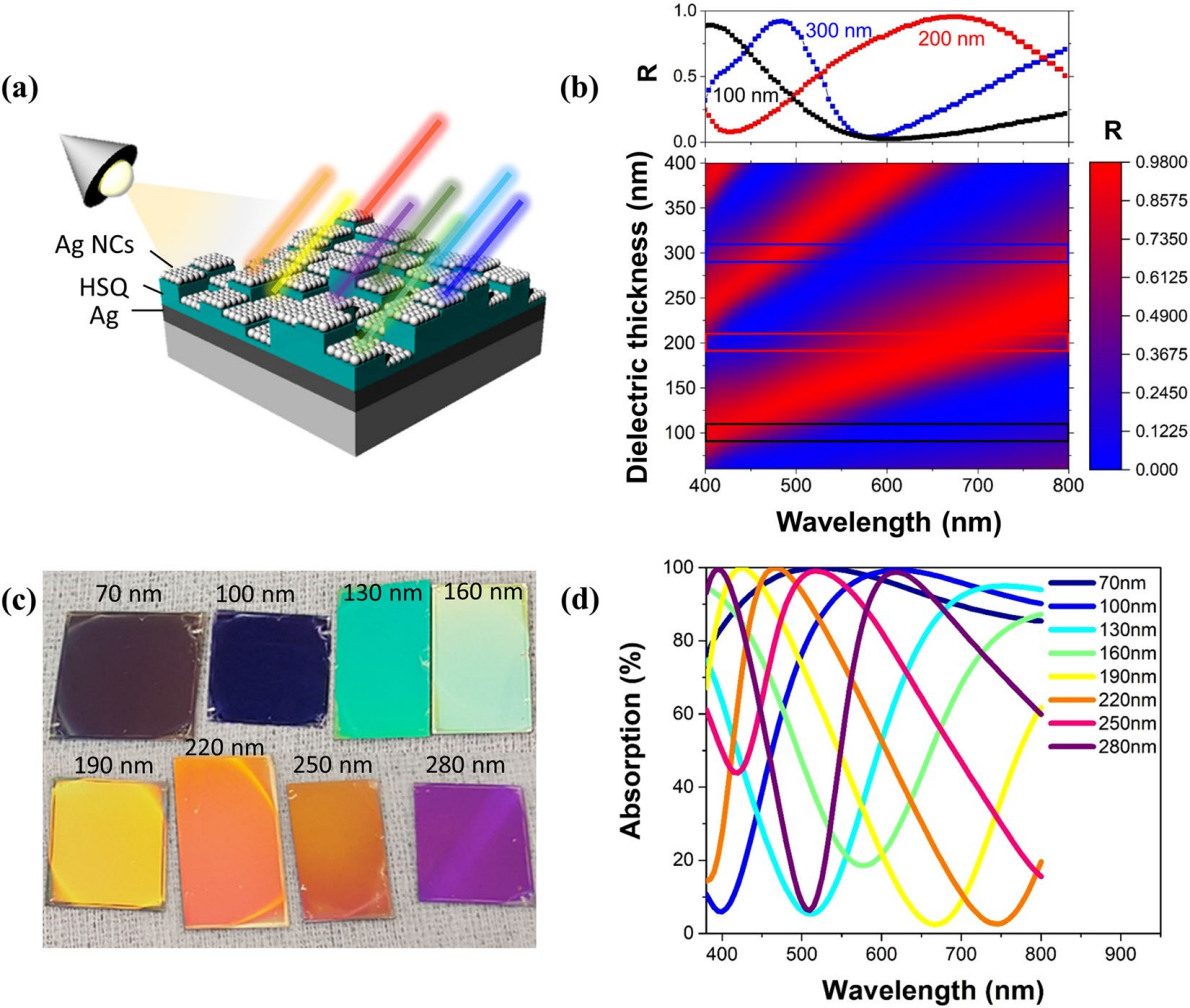
(**a**) Schematic of the multi-level F–P absorber for highly integrated reflective vivid colour generation. (**b**) FDTD simulation of the reflectance as a function of the wavelength and HSQ cavity thickness in the Ag-NC-based absorber. (**c**) Photograph of the absorbers with eight different colours including blue, cyan, green, yellow, orange, magenta, wine, and purple. (**d**) Measured reflectance spectra of the absorbers with different HSQ cavity thicknesses of 70 to 280 nm.

The representative geometry of the multi-structural F–P cavity is illustrated in Fig. 1(a). This structure, formed by the simultaneous introduction of cavity layers having various thicknesses, exhibits different reflection colours depending on the thickness of the layers. For the design of our asymmetric F–P cavity structures, the lossless hydrogen silsesquioxane (HSQ) (Supplementary Fig. S1), which has optical properties similar to those of SiO_2_, was used for the cavity layer and a deposited Ag thin film (100 nm) was used as the bottom mirror. We intentionally chose a porous metallic Ag layer with a high optical loss as the top layer for a broadband light absorption. To calculate the reflectance of the F–P cavity absorber with the porous Ag NC film, we used the full-wave finite-difference time-domain (FDTD) method. For this simulation, the complex refractive indices of the porous Ag NC film and HSQ cavity were obtained by an ellipsometry analysis and data reported by Palik^33^ were employed for the bottom Ag.

The calculated reflectance is plotted in Fig. 1(b), as a function of the HSQ cavity thickness and incident wavelength for the Ag-NC–HSQ–Ag absorber having a Ag NC film thickness of 30 nm. The reflectance peak continuously red-shifted from 400 to 800 nm as a function of the cavity thickness in the range of 60 to 400 nm, covering the entire visible range. The second reflective peak was generated when the cavity thickness was larger than 210 nm. The black, red, and blue horizontal boxes on the colour map present the reflectance spectra at dielectric thicknesses of 100, 200, and 300 nm, respectively. Notably, the reflection dip value was almost zero in a broad wavelength range, showing that the high reflection peak and very small reflection bandwidth could provide vivid colours. The broadband absorption is attributed to the porous metallic Ag layer, which was a homogeneous mixture of metal and pores, unlike the bulk Ag. The dielectric properties of the porous Ag film were compared with those of the Ag bulk using spectroscopic ellipsometry (Supplementary Fig. S2). The properties of the macroscopic metallic Ag material were derived using the effective medium approximation. In the visible–near-infrared (NIR) range, the real part of the complex dielectric function was less negative and less steeply sloped than that of the deposited Ag and the imaginary part of the function, which represents the optical loss, was higher. This demonstrates that the high optical loss of the porous metallic surface layer improved the absorption.

The fabricated large-area F–P cavity absorbers with eight different cavity thicknesses exhibited high saturations and bright colours, including purple, blue, cyan, light green, yellow, light orange, orange, and magenta, as shown in Fig. 1(c). The absorbance properties of the eight F–P cavities were measured using a Varian’s Cary 5000 ultraviolet (UV)–visible–NIR spectrometer. The absorption (*A*) was simply calculated as *A* = 1 − *R* − *T*, where *R* and *T* are the measured reflectance and transmittance, respectively. The transmittance (*T*) of the absorber was zero. Compared with those of the bulk-Ag-film-based absorbers^34^, which had wide reflection bands, the proposed F–P absorber had an excellent reflective spectral selectivity with a small bandwidth and high intensity owing to its broadband high light absorption, as shown in Fig. 1(d). According to our previous study^32^, the full width at half maximum of the bulk-Ag-film-based absorber was only 28 nm, while that of the Ag-NC-based absorber was approximately 300 nm for the same thickness of the dielectric layer. The increase in thickness of the cavity layer led to red shift of the cavity resonance wavelength, which could cover the entire colour spectrum corresponding to the visible region (400–800 nm).

### Fabrication of a stepwise Ag-NC-based F–P absorber

**Figure 2:**
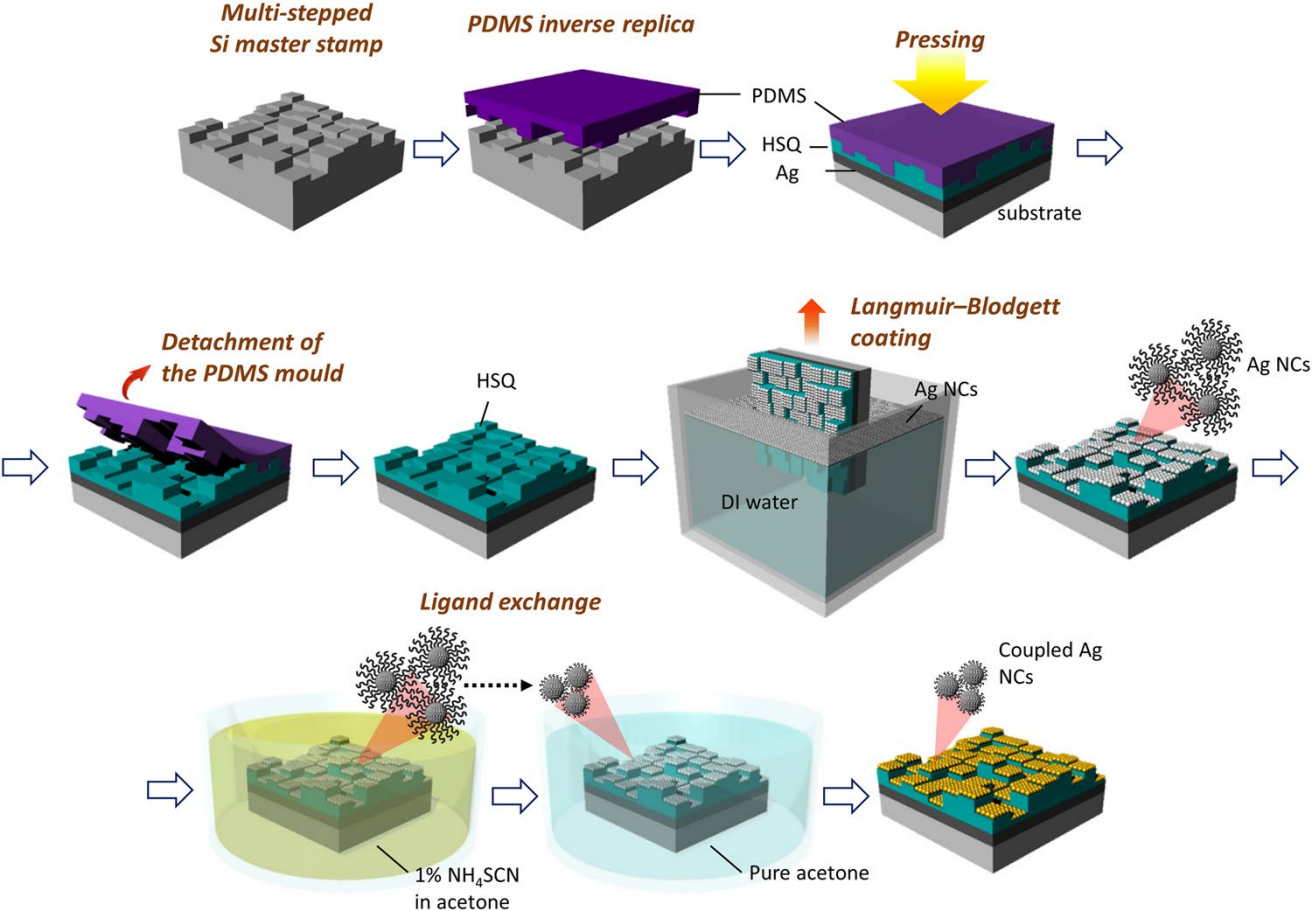
Schematic of the fabrication of the Ag-NC-based multi-absorber using the nanoimprint and NC coupling processes.

To experimentally evaluate the proposed F–P cavity absorber, we simultaneously fabricated multi-layered structures (Ag NC/HSQ/Ag) with eight different cavity thicknesses. Figure 2 shows a schematic of the overall manufacturing process. To perform the nanoimprint lithography, a Si master stamp with microscale pixel arrays with eight different heights was designed by repeating the photolithography and reactive-ion etching (RIE) processes three times (Supplementary Fig. S3). An eight-layered soft replica mould was then produced by moulding with polydimethylsiloxane (PDMS), which is a flexible elastomeric polymer. The pattern of the stamp was fabricated by replicating a hard Si master stamp in reverse.

We used the HSQ resin, a derivative of SiO_2_, as the dielectric material of the F–P cavity. To fabricate a multi-level pixelated HSQ layer, the HSQ resist was spin-coated onto the eight-layered PDMS mould and filled in the micropatterns. The HSQ layer was then transferred to the Ag deposited silicon substrate by applying pressure. Subsequently, the standard processes used to obtain amorphous SiO_2_ from HSQ, including the removal of the solvent by soft baking on a hot plate, were performed. The HSQ-curing step was performed in a UV-ozone-cleaner chamber. After the HSQ multi-level patterned layer was fabricated, the Ag NC film was uniformly coated onto the three-dimensional (3D) micropatterned HSQ dielectric layer using the Langmuir–Blodgett (L–B) method. The substrate was dipped vertically through the well-arranged NC film floating on the surface of the water.

For the preparation of our unique Ag NC top layer of the absorber, an octane-based oleylamine (OLA) ligand-capped Ag NC dispersion was coated onto the substrate. The NCs had diameters of approximately 10 nm and were spaced 2 nm apart owing to the long OLA ligands. The OLA-capped NC film sample was immersed in a 1% ammonium thiocyanate (NH_4_SCN) solution and the OLA ligand was replaced with 0.2-nm short thiocyanate (SCN) ligands to electrically couple the NCs to each other^32,35,36^.

**Figure 3:**
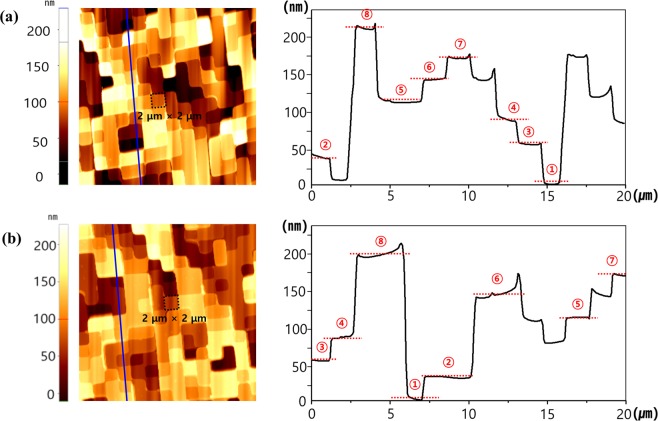
Typical two-dimensional atomic force microscopy (AFM) surface profiles (Scan size was 20 × 20 μm) and eight-level height profiles along the blue lines in the AFM images: (**a**) silicon master stamp and (**b**) patterned HSQ multi-layer.

To confirm the results for the processed stepwise HSQ layer, the structural information for the Si master stamp and HSQ layer was compared using AFM (Park Systems, XE-100). The 2 × 2 μm square pixels included in the Si stamp were randomly arranged, as shown in the surface profile image in Fig. 3(a). In the area indicated by the blue solid line, the sidewall profile of the micropixels shows various heights, from 0 to 210 nm in steps of 30 nm. The HSQ multi-layer was clearly defined in the eight layers, from the lowest to the highest (2 × 2 μm pixels), not including the residual layer, as shown in Fig. 3(b). Furthermore, the boundaries of the micropixel were clear and not tapered, and the surface of the HSQ pixel was not rough (root-mean-square roughness < 0.581 nm, Supplementary Fig. S4). These results indicate that the nanoimprinted stepwise HSQ dielectric layer is suitable for the realisation of simultaneously integrated reflective multiple colours.

**Figure 4:**
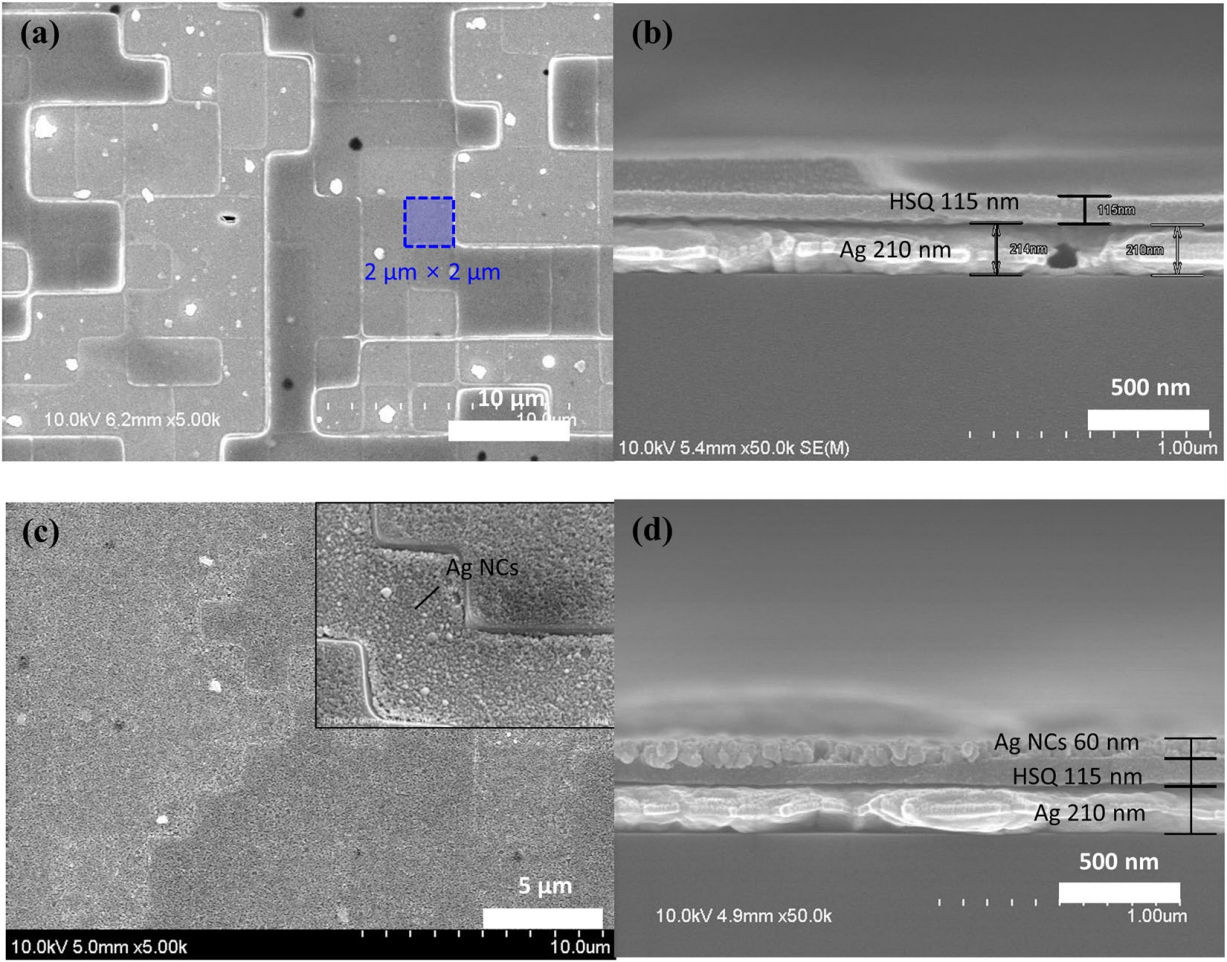
(**a**,**c**) Top-view and (**b,d**) cross-sectional SEM images of the multi-stepped HSQ layer and Ag-NC-based multi-level absorbers used for the generation of various colours, respectively.

The HSQ layer with the residual layer has a uniform and smooth surface, as shown in the SEM images in Fig. 4(a,b). The residual layer was adjusted according to the HSQ concentration to determine the desired minimum HSQ thickness. The porous Ag NC layer, transparent to light, was uniformly coated on the HSQ layer using L–B coating methods (Fig. 4(c,d)). The thickness of the NCs was approximately 68 nm, which was the same for all the eight layers. It is challenging to coat NCs over the entire surface of the substrate by other coating methods such as dip coating and spray coating (Supplementary Fig. S5). When the NCs were spin-coated, a smaller HSQ thickness led to thicker NCs, as shown in Supplementary Fig. S6. The porous metallic film consisted of Ag NCs connected by short and conductive ligands through the solid–ligand exchange reaction.

### Simultaneous implementation of micropixelated vivid colours

**Figure 5:**
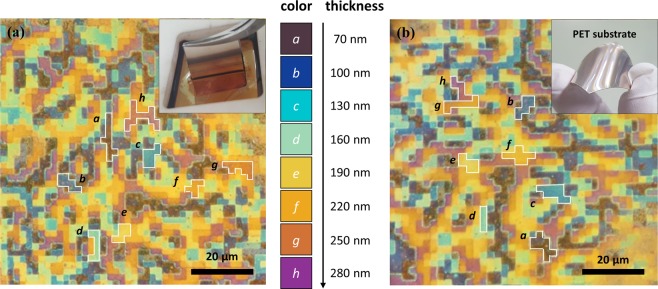
Optical microscopy images of the reflective colours obtained by the eight-level multi-absorber with 2 × 2 μm square pixels on a (**a**) rigid silicon wafer and (**b**) PET flexible substrate.

Through the nanoimprint patterning and NC solution process, we successfully integrated the eight colours with high-resolution pixels (2 × 2 μm) on only one substrate. Figure 5(a) shows an optical microscopy image of the pixelated multi-colours of the absorber sample with dimensions of 1 × 1 cm. The vivid colours, from dark blue to magenta, indicated by the white solid line (*a-h*) in the image, were realised by the HSQ layer with thicknesses of 70–280 nm, including the 70-nm residual layer. The pixelated reflection colours were the same as the large-area colours in Fig. 1(c). In particular, the interfaces of the different colours were clear and perfect without colour mixing. The nanoimprint lithography and NC solution process, which enable a large-scale processing at room temperature, can be used in novel applications as they can be applied to mechanically flexible substrates. Therefore, we implemented multi-colour pixels on a flexible polyester terephthalate (PET) film with an area of 1 × 1 cm, as shown in Fig. 5(b).

**Figure 6:**
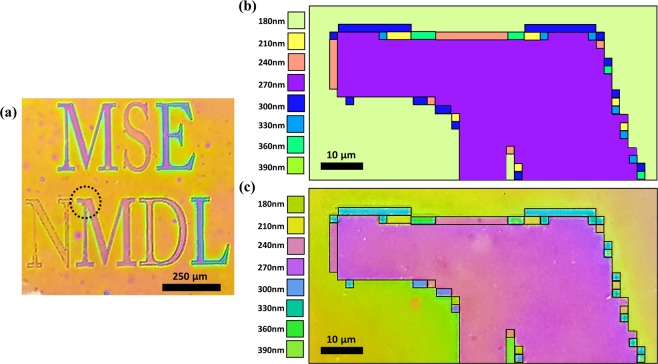
(**a**) Optical microscopy image (magnification of 50) of the fabricated colour signature of the laboratory. (**b**) Calculated enlarged image and (**c**) optical microscopy image (magnification of 1000) of a colour signature exhibiting bright colours with a high contrast.

To demonstrate the application feasibility of the proposed concept for a simultaneous high-resolution multi-colour printing of arbitrary images, we designed the signature of our laboratory, which was formed using absorbers with various dimensions, from 2 × 2 μm to 40 × 40 μm, set at different positions. The optical microscopy observation confirmed the fabricated colourful ‘MSE NMDL’ letters at a magnification of 50 (Fig. 6(a)). ‘M’ in ‘NMDL’ was designed to have partially different colours. The colours were calculated considering the eight different thicknesses of the dielectric layer, from 180 to 390 nm, as shown in Fig. 6(b). The different colours of the letters well matched the calculated colours (Fig. 6(c)), which confirms the clear reflective colours in a small area. This could be used for high-resolution display applications with submicrometre pixels.

**Figure 7:**
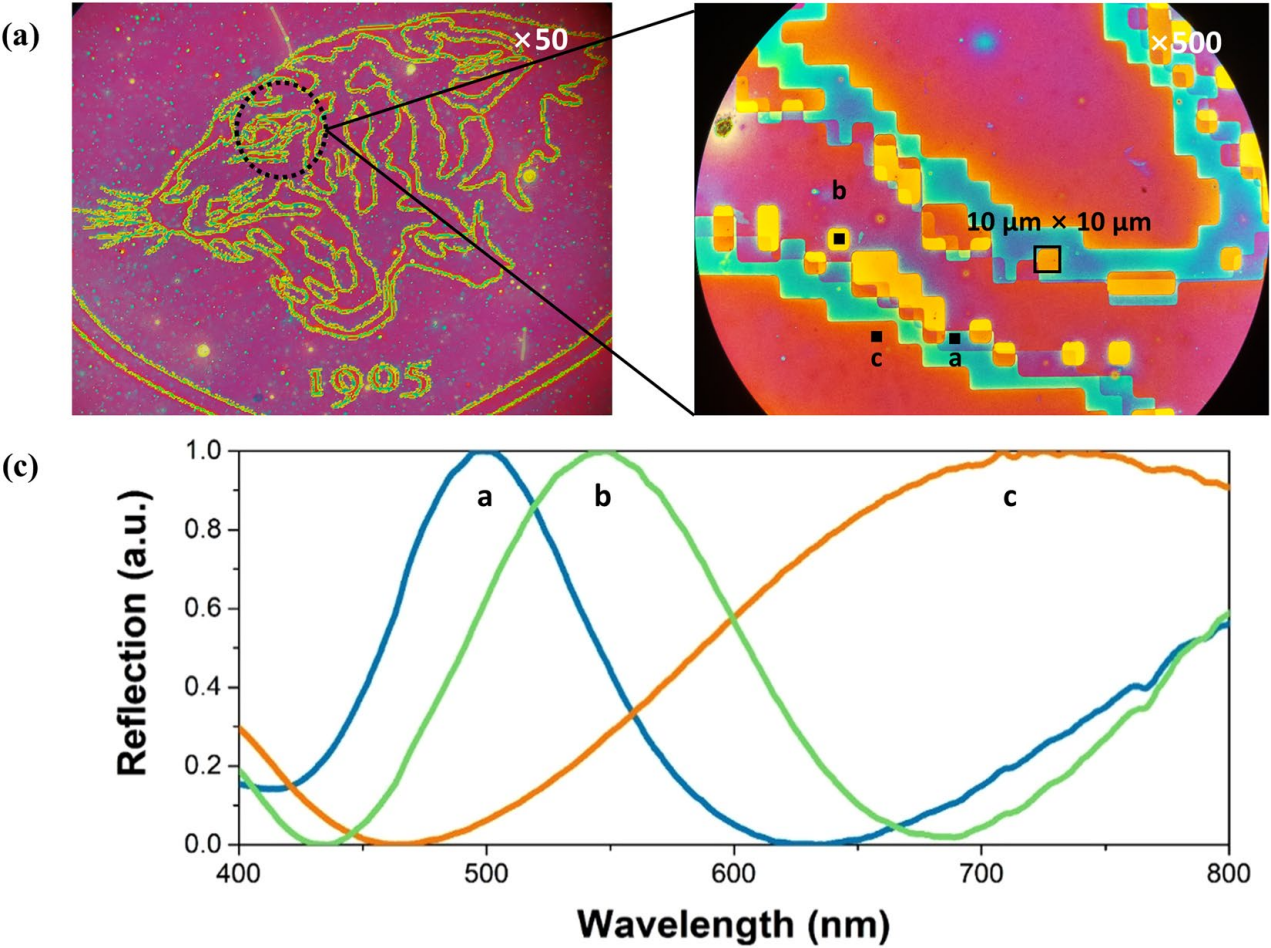
(**a**) Optical microscopy images (magnifications of 50 and 1000) of a colour signature of Korea University. (**b**) Reflection spectra of the reflective colours obtained with square pixels with dimensions over 10 μm measured by a UV–visible microspectrometer.

As shown in the enlarged tiger’s eye (10 × 10 μm pixels) signature of Korea University in Fig. 7(a), the highly reflective colours were generated and the boundaries where one colour ends and another begins were clearly defined. The three localised parts of the pixelated absorbers (square pixels with a, b, and c points) exhibited a high colour purity, according to the microspectrometer measurements (Fig. 7(b)). The high-resolution colour images demonstrate that the nanoimprint process is a promising nanofabrication method to produce images including various scales, from one to one hundred micrometres, by simultaneously realising different cavity thicknesses and areas. The multi-absorber based on the NC solution process can easily realise a passive colour display with high-resolution pixels over a large area. Furthermore, if a phase-change material is introduced to the dielectric layer, active reflective colours, which actively operate according to electric field or heat, may be realised.

## Discussion

Various specific colours obtained by submicrometer pixels were simultaneously realised through the stepwise F–P absorber. The eight-stepped dielectric layer was thoroughly designed and fabricated using the polymer resin by the optimised nanoimprinting method. The nanoimprint lithography provided a simple solution to fabricate a high-resolution pixel on a large-area, regardless of the substrate. With the aid of the highly lossy porous Ag, the colour purity was improved owing to the reflection of almost zero, in conjunction with the small bandwidth. We expect that the vivid colours based on the F–P multi-absorber can be applied for the development of high-resolution displays and colour printing.

## Methods

### Fabrication of multi-cavity layers using nanoimprinting

For the simultaneous fabrication of the stepped dielectric layer, a silicon master stamp of pixelated multi-layers was prepared. The silicon master stamp was designed to have randomly square size and height. Three-step photolithography and etching sequences were carried out to fabricate the pixels with eight different thicknesses^37^. In the first step, the negative photoresist was spin-coated onto a uniform 1 μm-thick film of Si. After the UV exposure during the pattern transfer, the resist was developed, and then dried under a nitrogen atmosphere. The resist-patterned wafer was then immediately coated by e-beam evaporation with nickel to generate a 100 nm-thick photomask. After the removal of the nickel-coated resist regions using a 1-methyl-2-pyrrolidone solution, only the remaining stripes of nickel served as a photomask for the subsequent RIE. After this step, an etched Si stamp was fabricated using photolithography and RIE as the second step (etching depth: 60 nm). After the second step, the depths of the Si stamp were 0, 30, 60, and 90 nm. After the third processing step, the Si stamp had eight layers with depths of 0, 30, 60, 90, 120, 150, 180, and 210 nm (etching depth: 120 nm). After the RIE, the photomask was removed in a solution containing ceric ammonium nitrate, nitric acid, and deionised water. The pixelated multi-layer Si stamp was then rinsed in deionised water and dried under a nitrogen atmosphere. The PDMS mould was prepared by pouring a mixture of Sylgard 184 A and curing agent Sylgard 184B over the eight-layer Si wafer. After the pouring, the mixed solution was degassed for 10 min and cured at 80 °C for 1 h. Subsequently, the cured PDMS mould was detached from the master Si stamp. The HSQ resin was prepared by dissolving a flowable oxide (FOX-16) purchased from Dow Corning Inc. in methyl isobutyl ketone. The HSQ solution was spin-coated onto the PDMS mould at 3000 rpm for 30 s. The PDMS mould with the HSQ layer was immediately placed in contact with the deposited Ag substrate. The HSQ patterning process was completed by applying a pressure of 500 kPa for 5 min. The PDMS mould was then detached from the eight-layer HSQ patterns.

### Chemically engineered lossy metallic layer as the top layer

To form the porous Ag NC material, the L–B deposition technique was used with a 5 wt% Ag-NC–octane dispersion solution. This method provided uniform NC multi-layers. For the assembly of the NCs, a Teflon trough was carefully cleaned and filled with distilled water. The well-dispersed Ag NCs were carefully spread on the water surface drop-by-drop using a glass syringe, and then the NC multi-layer was left for approximately 30 min. The NC multi-layer was transferred to the substrate deposited with the HSQ multi-layer during the compression by vertically dipping the substrate into the trough and slowly pulling it up (1 mm/s). The coated NC layer was dried on a hot plate at 90 °C for stabilisation. To couple the Ag NCs through the short ligands, the NC-coated sample was immersed in a 1% NH_4_SCN acetone solution for 1 min for ligand replacement (OLA replacement by SCN ligands). The sample was then transferred to a bath of pure acetone to wash away the excess ligand.

### Measurement of reflective and absorption properties

The refractive indices (*n*) and extinction coefficients (*κ*) of the porous Ag film and HSQ dielectric material were obtained by spectroscopic ellipsometry (M-2000D ellipsometer, J.A. Woollam Co.). The measurements were carried out in the wavelength range of 192–1654 nm at an angle of incidence of 75°. The samples were prepared by coating on the silicon wafer for the analysis. The physical quantities were obtained by comparing the measured data (*ψ*, *Δ*) with the theoretically calculated values using the CompleteEASE software package.

The reflectance and absorption of the F–P absorber were measured using a Cary 5000 UV–visible–NIR spectrophotometer without polarisation, at a spectral band-pass of 1 nm in the wavelength range of 400–800 nm. The deposited 200 nm-thick Ag mirror was used for the reflectivity baseline of 100%. We used a UV–visible microspectrometer (CRAIC) for the measurement of the reflectance spectrum of the few micropixels.

## Supplementary information

Supplementary Info
