# Supplementary material for: Generation of highly integrated multiple vivid colours using a three-dimensional broadband perfect absorber
Source: Sci Rep. 2019 Oct 16;9:14859. doi: 10.1038/s41598-019-49906-3 (PMC6795891; doi:10.1038/s41598-019-49906-3)
Supplement: Supplementary file 1 — Supplementary Info [file 41598_2019_49906_MOESM1_ESM.pdf]

Supplementary Information

Generation of highly integrated multiple vivid colors using a three-dimensional broadband perfect absorber

Soo-Jung Kim [1], Pil-Hoon Jung [1], Wonjoong Kim [1], Heon Lee [1,] and Sung-Hoon Hong [2,*]*

[1] Department of Materials Science and Engineering, Korea University, Anam-dong 5-1, Sungbuk-Ku, Seoul 136-701, Republic of Korea.

[2] ICT Materials & Components Research Laboratory, Electronic and Telecommunications Research Institute Gajeong-dong, Yuseong-gu, Daejeon 305-700, Republic of Korea.

Corresponding Authors

E-mail: shong@etri.re.kr, heonlee@korea.ac.kr

This document provides supplementary information to "Generation of highly integrated multiple vivid colors using a three-dimensional broadband perfect absorber". The supplementary information is 6 pages long, and contains 6 figures.

Part 1.

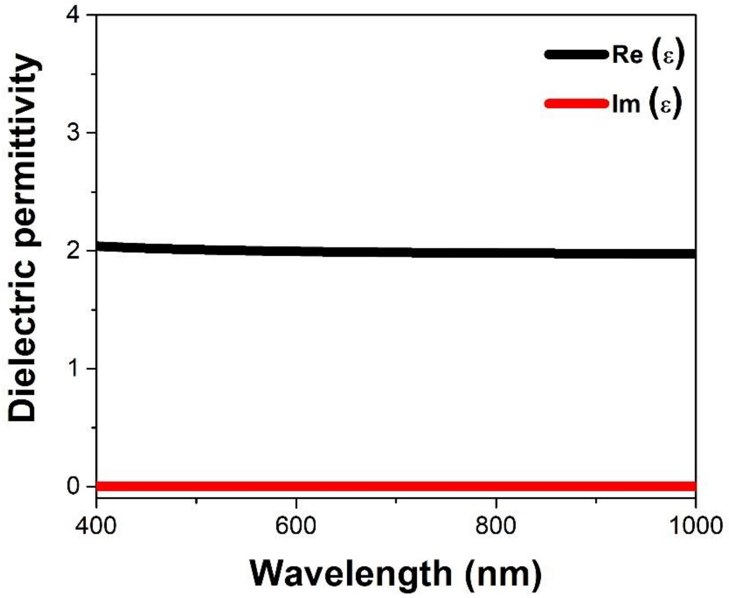

Fig. S1. Real part and imaginary part of dielectric constant ($\varepsilon = \varepsilon' + i\varepsilon''$) of lossless HSQ dielectric layer.

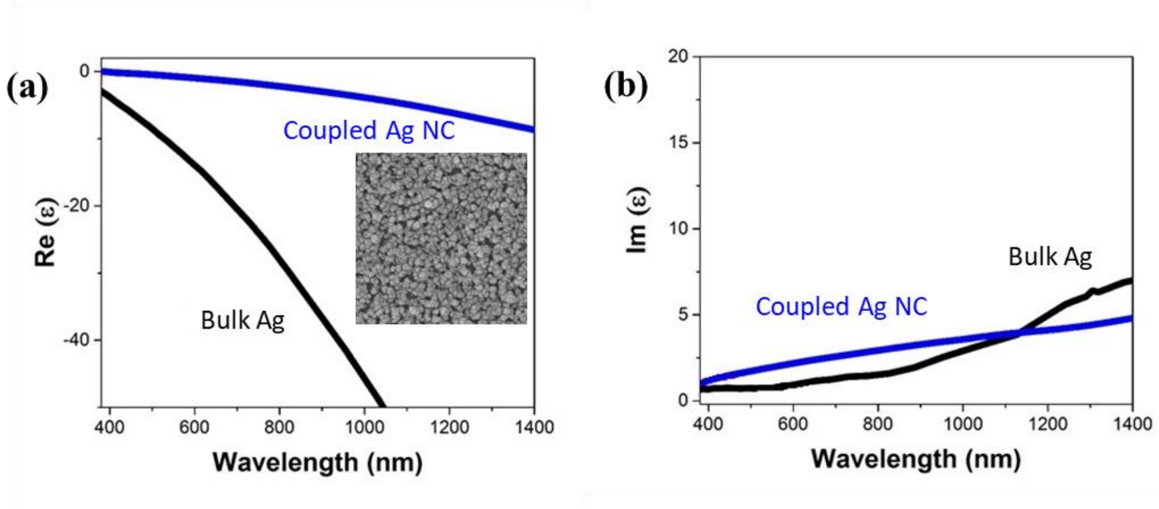

Fig. S2. (a) Real part and (b) Imaginary part of dielectric constant ($\varepsilon = \varepsilon' + i\varepsilon''$) of the coupled Ag NC film and bulk Ag film.

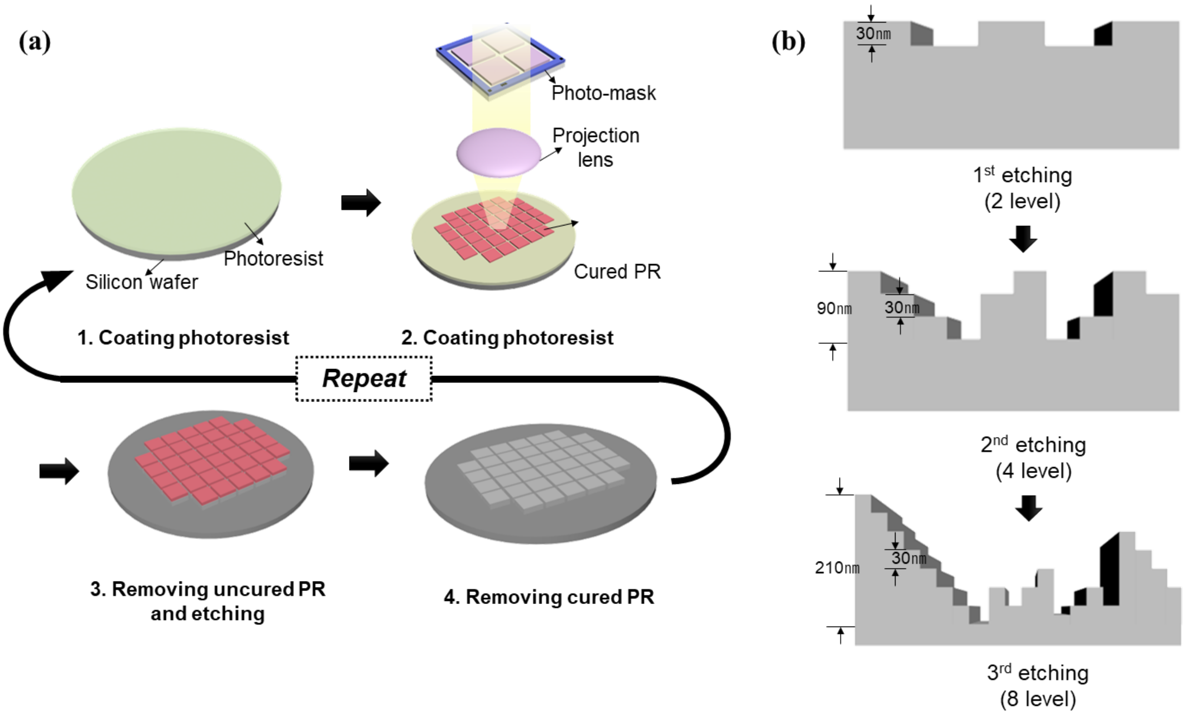

Fig. S3. (a) Schematic of multi-stepped silicon master stamp using photolithography and etching process with 3-steps. (b) Results from step-by-step photolithography and etching process.

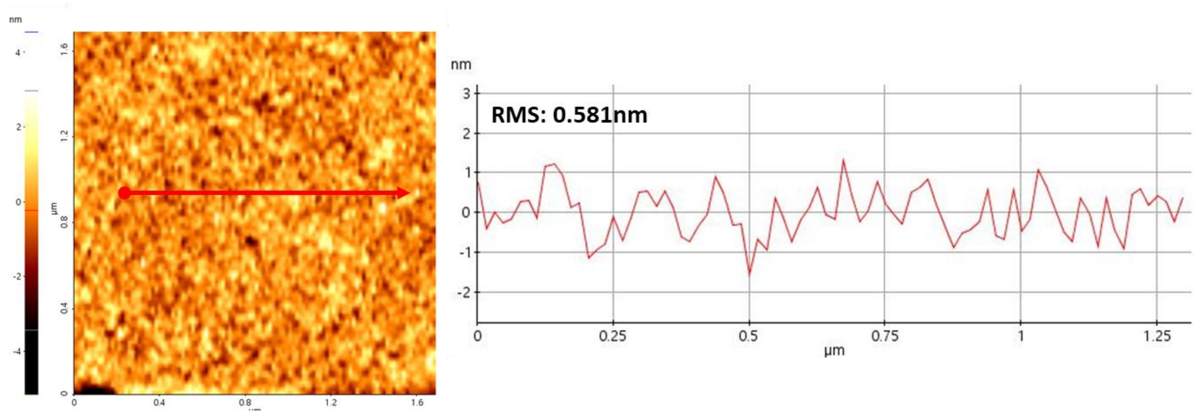

Fig. S4. The roughness information of the pixelated HSQ surface

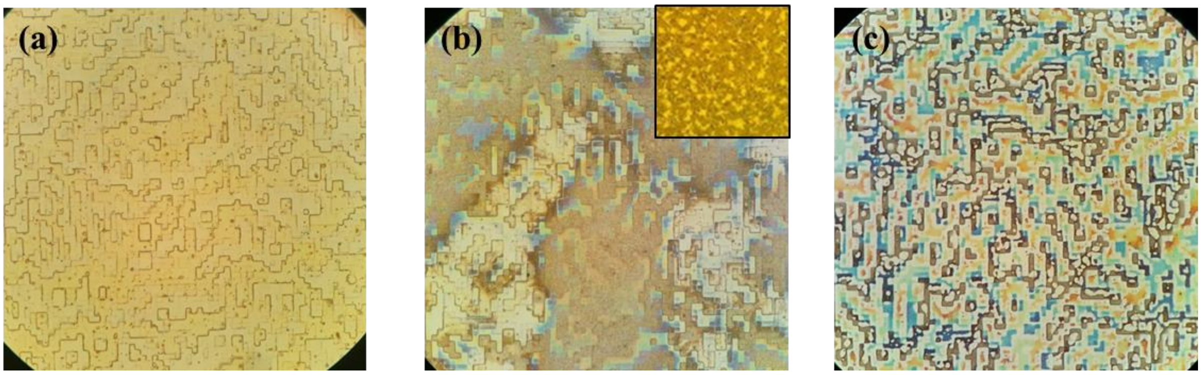

Fig. S5. Results of multi-absorber fabrication with various nanocrystal coating methods: (a) Dip coating, (b) Spray coating, and (c) Spin coating

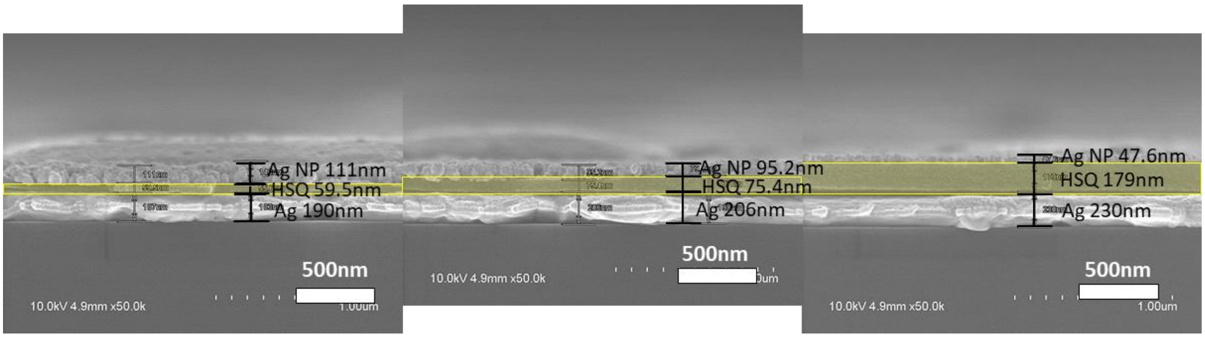

Fig. S6. Using spin-coating method: SEM cross section of the absorber with different Ag NC thickness due to the difference of the HSQ thickness.
